# Genome scale prediction of substrate specificity for acyl adenylate superfamily of enzymes based on active site residue profiles

**DOI:** 10.1186/1471-2105-11-57

**Published:** 2010-01-27

**Authors:** Pankaj Khurana, Rajesh S Gokhale, Debasisa Mohanty

**Affiliations:** 1 National Institute of Immunology, Aruna Asaf Ali Marg, New Delhi, India; 2 Current address: Defence Institute of Physiology and Allied Sciences, Delhi, India

## Abstract

**Background:**

Enzymes belonging to acyl:CoA synthetase (ACS) superfamily activate wide variety of substrates and play major role in increasing the structural and functional diversity of various secondary metabolites in microbes and plants. However, due to the large sequence divergence within the superfamily, it is difficult to predict their substrate preference by annotation transfer from the closest homolog. Therefore, a large number of ACS sequences present in public databases lack any functional annotation at the level of substrate specificity. Recently, several examples have been reported where the enzymes showing high sequence similarity to luciferases or coumarate:CoA ligases have been surprisingly found to activate fatty acyl substrates in experimental studies. In this work, we have investigated the relationship between the substrate specificity of ACS and their sequence/structural features, and developed a novel computational protocol for *in silico *assignment of substrate preference.

**Results:**

We have used a knowledge-based approach which involves compilation of substrate specificity information for various experimentally characterized ACS and derivation of profile HMMs for each subfamily. These HMM profiles can accurately differentiate probable cognate substrates from non-cognate possibilities with high specificity (Sp) and sensitivity (Sn) (Sn = 0.91-1.0, Sp = 0.96-1.0) values. Using homologous crystal structures, we identified a limited number of contact residues crucial for substrate recognition i.e. specificity determining residues (SDRs). Patterns of SDRs from different subfamilies have been used to derive predictive rules for correlating them to substrate preference. The power of the SDR approach has been demonstrated by correct prediction of substrates for enzymes which show apparently anomalous substrate preference. Furthermore, molecular modeling of the substrates in the active site has been carried out to understand the structural basis of substrate selection. A web based prediction tool http://www.nii.res.in/pred_acs_substr.html has been developed for automated functional classification of ACS enzymes.

**Conclusions:**

We have developed a novel computational protocol for predicting substrate preference for ACS superfamily of enzymes using a limited number of SDRs. Using this approach substrate preference can be assigned to a large number of ACS enzymes present in various genomes. It can potentially help in rational design of novel proteins with altered substrate specificities.

## Background

**Figure 1:**
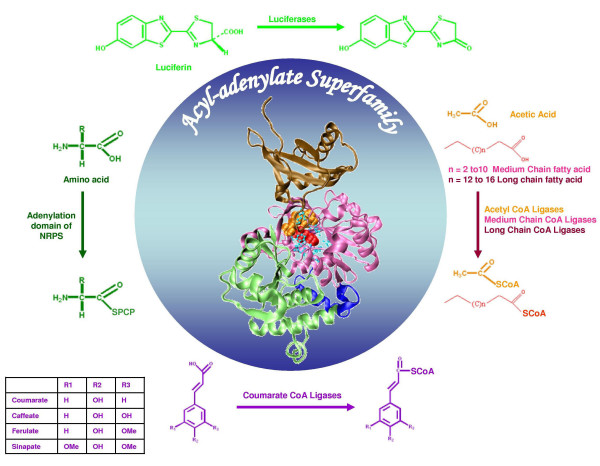
**Schematic representation of the acyl-adenylate superfamily and its various subfamilies**. The figure shows the chemical structure of the substrates and products for the reactions catalyzed by members of each subfamily. The various subfamilies utilize different carboxylic acid substrates and transfer acyl moiety to either CoA or enzyme bound phosphopantetheine arm. The luciferase catalyzes conversion of luciferin to oxyluciferin. All the subfamilies are known to take up a similar 3-dimensional fold (depicted in centre) which has a large N-terminal domain and a small C-terminal domain. The structure shown is the adenylation domain of gramicidin synthetase (PDB code: 1AMU). The cofactor AMP (orange) and substrate Phenylalanine (red), shown in CPK, bind in the cleft separating the two domains. The substrate binding residues are shown in cyan as ball and stick.

The acyl:CoA synthetases belong to the "AMP-forming superfamily" or "acyl-adenylate/thioester-forming" superfamily. ACS catalyze transfer of a wide variety of acyl, aryl and aminoacyl moieties from their corresponding acids to the phosphopantetheine group of coenzyme A (CoA) or other carrier proteins through formation of a thioester bond. Based on the type of the carboxylate substrate, enzymes of this superfamily have been functionally divided into several subfamilies, namely acetyl:CoA synthetases (AcCS, EC 6.2.1.1), medium chain:CoA synthetases (MCS, EC 6.2.1.2), long chain:CoA synthetases (LCS, EC 6.2.1.3), 4-coumarate:CoA ligases (4CL, EC 6.2.1.12), luciferases (EC 1.13.12.7) and adenylation domains of NRPSs (Non-Ribosomal Peptide Synthetases) (Figure 1). AcCS activates C_2_-C_4 _fatty acids, MCS activates C_4_-C_12 _and LCS activates C_10_-C_22 _fatty acids. Luciferases utilize luciferin as the substrate. 4CLs catalyzes the activation of various cinnamic acid derivatives (cinnamate, coumarate, caffeate, sinapate, ferulate etc.) [1]. The substrates for adenylation domains of NRPS include various proteinogenic as well as non-proteinogenic amino acids. The enzymes carry out a two-step reaction process which proceeds through the hydrolysis of pyrophosphate (Additional file 1, **Figure S1**). In the first step of the reaction, ATP in presence of Mg^2+ ^reacts with a carboxylate substrate to form an acyl-adenylate intermediate with the simultaneous release of pyrophosphate. In the second step of the reaction, the acyl-adenylate intermediate is esterified to CoA in case of AcCS, LCS, MCS, 4CL subfamily while adenylation domains of NRPS catalyze the transfer of the acyl adenylate to 4'-phosphopantheteine group of Peptidyl Carrier Protein (PCP). In case of luciferases the acyl intermediate is oxidized by molecular oxygen. Since the enzymes form thioester derivatives through an acyl-adenylate intermediate, this superfamily is also known as "acyl-adenylate/thioester-forming" superfamily. Apart from FACL (Fatty Acyl CoA Ligases), which transfer the acyl-adenylate intermediate to CoA, recently a new family of enzymes called FAAL (Fatty Acyl AMP Ligases) which transfer acyl adenylate to carrier protein domains of adjacent NRPS/PKS clusters have been discovered in *Mycobacterium tuberculosis *[2,3].

Acyl:CoA synthetases have been characterized from both prokaryotic and eukaryotic organisms and are found in a wide variety of tissues and cell organelles. Acyl:CoA derivatives are known to play important role in cell signaling [4,5], post-translational protein modification [6,7], intracellular protein transport [8] and transcriptional control of genes involved in lipid metabolism [9,10]. NRPSs catalyze the synthesis of a large number of pharmacologically important peptides eg., vancomycin, cephalosporins, cyclosporins, penicillins [11]. 4-Coumarate:CoA ligases play central role in the biosynthesis of phenylpropanoid-derived compounds like coumarins, stilbenes, and lignin, which are important in plant growth and development [12]. Since the members of the superfamily are involved in synthesis of siderophores like mycobactin and yersiniabactin in *M. tuberculosis *and *Y. pestis*, which are important for microbial virulence; several studies have implicated these enzymes as important drug targets [13]. The enzymes of this superfamily have also been targets of interesting protein engineering studies. Eg. The acyl:CoA synthetases have been used to incorporate unusual acyl groups into a variety of secondary metabolites, e.g. antibiotics. This helps in the synthesis of novel antibiotics with improved potency. The adenylation domains of NRPSs can be engineered to generate novel peptide antibiotics. Luciferase is extensively used for *in-vivo *luminescence monitoring [14] and also as reporter of gene expression and regulation [15,16]. The members of the superfamily are very diverse in their primary sequence and the overall sequence similarity ranges between 20-30%. However there are reports of presence of highly conserved motifs within the superfamily, especially the putative "AMP-binding domain signature motif" (PROSITE PS00455) ([LIVMFY] - {E} - {VES} - [STG] - [STAG] - G - [ST] - [STEI] - [SG] - x - [PASLIVM] - [KR]). The presence of this motif is used as the main criteria to group these enzymes in one superfamily [17].

Crystal structures have been elucidated for several members of the superfamily, some with and some without substrates [18-30]. Inspite of large differences in their primary sequences (similarity ranging from 15%-45%), the enzymes adopt a conserved structural fold with large N-terminal and small C-terminal domain with the active site lying between the interface of the two domains (Figure 1). The three-dimensional structures in complex with substrates have allowed identification of the substrate binding pocket. The crystal structure of adenylation domain of gramicidin synthetase (PheA) has helped in identification of 10 amino acids forming the substrate (phenylalanine) binding pocket (PDB code: 1AMU). The hypothesis that the amino acids forming the substrate binding pocket can potentially be the specificity determining residues was established by the fact that the changes in these residues have directly altered the substrate specificity of peptide synthetases [31-33]. For adenylation domains of NRPSs, it has been shown that prediction of substrate specificity based on the active site pocket residues are more accurate than whole sequence comparisons [31,32,34]. Similarly, putative substrate binding pocket residues have been identified for 4CLs and luciferases and their mutations have successfully altered the substrate specificity profiles of these enzymes [1,35-41].

Recently there have been reports of enzymes which show "anomalous" substrate specificity. CG6178 from *Drosophila melanogaster *shows maximum sequence similarity to luciferase. However biochemical experiments show that it is a long chain fatty acyl:CoA synthetase and does not function as luciferase [42,43]. Similarly two proteins from *Arabidopsis thaliana*, namely At4 g05160 and At5 g63380 are annotated as 4CL-like proteins in the databases. However biochemical analysis revealed them to be utilizing long chain fatty acids as the substrates [44]. Another example is At1 g62940 which shows similarity to 4CL but is experimentally found to be a medium chain CoA ligase and has been shown to be involved in pollen development and sporopollenin biosynthesis [45,46]. It is unclear how these enzymes show high sequence similarity to one subfamily but have substrate preference corresponding to other subfamily. A large number of genes belonging to acyl adenylate superfamily have been deposited in public databases without any substrate preference being assigned to them. Predicting the substrate preference of these enzymes will help in unravelling their specific biological functions.

In this manuscript, we have attempted to develop a novel computational approach which will be fast enough for genome scale prediction of substrate specificity of this biologically important enzyme family. We have used a knowledge-based approach which combines substrate specificity information from experimentally characterized members of this superfamily with structural information from available crystal structures to derive predictive rules for correlating sequence to substrate specificity. Based on detailed sequence and structural analysis we have identified residues which play a crucial role in dictating substrate preference of this enzyme superfamily. We also demonstrate that, based on profiles of such specificity determining residues it is not only possible to predict the substrate specificities of various subfamilies accurately, but also rationalize the observed substrate preferences of enzymes which show anomalous substrate specificity. We have also carried out detailed structural analysis and ligand docking studies for each subfamily to understand the structural basis of substrate selection.

## Results And Discussion

### Phylogenetic and HMM profile analysis

The sequences belonging to various different subfamilies of acyl-adenylate superfamily were collected by keyword search and repeated BLAST searches [47] against the NR database of NCBI [48]. Only sequences with known substrate preference were catalogued. The resulting database comprised of 608 protein sequences with known substrate specificity (120 AcCS, 130 LCS, 119 4CL, 25 luciferases and 29 MCS). Since the adenylation domains are part of multidomain proteins, 190 adenylation domains with known substrates and correct domain boundaries were extracted from NRPSDB, a comprehensive database on domain arrangement and substrate specificity of NRPSs [34]. All-against-all pairwise sequence alignments were carried out for all 608 sequences to determine the similarity across the whole superfamily. As can be seen from Table 1 the similarity within the superfamily varies from 10-30%. Inter-subfamily similarity and intra-subfamily similarity values were overlapping i.e. a LCS may show higher percentage similarity to another LCS in the range of 10-30% and it may show similarity in the same range with luciferase also (Table 1). Hence for this superfamily of enzymes the substrate preference for an uncharacterized sequence cannot be assigned based on the closest sequence homolog.

**Table 1:** The range of pairwise sequence similarity between members of various subfamilies

Subfamily	LCS					
LCS (129)	10-30	MCS				

MCS (25)	10-20	20-50	4-CL			

4-CL (115)	3-30	10-30	20-90	Luciferase		

Luciferase (29)	10-30	10-30	10-35	50-90	AcCS	

AcCS (120)	5-30	10-30	4-30	10-30	10-90	NRPS

NRPS (190)	2-30	10-30	10-30	10-30	10-30	30-50

All against all pairwise sequence comparisons were carried out by Needleman and Wunsch program of the EMBOSS package. The figures in the parenthesis indicate the number of sequences included in our analysis for each subfamily.

**Figure 2:**
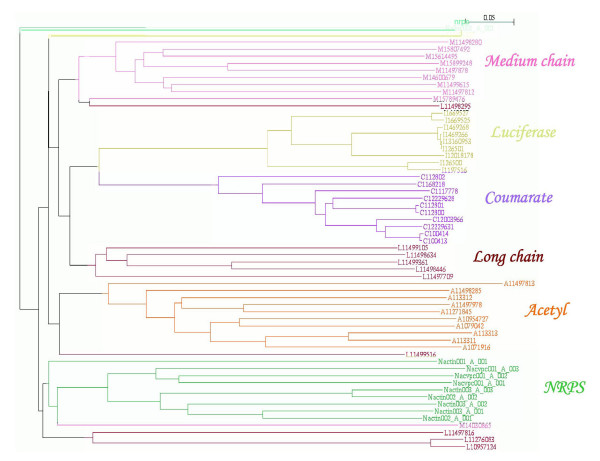
**A phylogenetic tree obtained from MSA of representative members of ACS superfamily**. The six subfamilies are represented in different colors. Each subfamily, namely AcCS (orange), 4CL (purple), MCS (pink), LCS (maroon), Luciferases (yellow), NRPS (green) cluster as separate groups in the dendrogarm.

Figure 2 shows a dendrogram obtained from a set of sixty representative sequences from various subfamilies. As can be seen, the sequences utilizing similar substrates have clustered together in the phylogenetic tree. This indicates that despite the large sequence divergence within each subfamily, the sequences of a given subfamily have distinct features which distinguish them from other subfamilies, such that they cluster in a subfamily specific manner. Therefore profile based methods like HMMs[49] can be used to devise a prediction protocol for assigning the substrate specificity to an uncharacterized protein belonging to acyl adenylate superfamily. For each subfamily, the sequences were randomly divided into training set and test set. The training set was used to derive subfamily specific HMM profiles and the test set was used to benchmark the prediction accuracy. The sequences of the training set were aligned by CLUSTALW and the resulting multiple sequence alignment was given as input to the HMMER package[49] for building HMM profiles for each subfamily. All the six HMM profiles were compiled in the form of a HMM library. Each sequence in the test set was matched with all six profiles in this library of HMM's using the program 'hmmpfam'. The test sequence was scored against each subfamily HMM and substrate preference was assigned to the test sequence based on best scoring HMM i.e. if the query sequence scores best with HMM for 4-Coumarate:CoA ligases, then the specificity assigned to the query would be Coumarate. By comparing predicted substrate specificity to the known substrate specificity, sensitivity (Sn) and specificity (Sp) were calculated to determine the overall prediction accuracy of the method. Table 2 summarizes the results for HMM based substrate specificity predictions for the acyl-adenylate superfamily. The sensitivity ranged from 0.91 for MCS to 1.0 for luciferases. The specificity was 0.96 for LCS, 0.99 for MCS and 1.0 for other subfamilies. Thus based on whole sequence HMM profiles it is possible to correctly group acyl adenylate superfamily of enzymes into various subfamilies as per their substrate specificity. However, this whole sequence HMM failed to predict correctly the substrate specificities for the enzymes, CG6178, At4 g05160, At5 g63380 and At1 g62940 which show sequence homology to luciferase and 4CLs, but utilize long chain or medium chain fatty acyl substrates (Table 3). Rather than predicting them as long chain or medium chain CoA synthetases, the HMM profiles predicted them as luciferases and 4CLs respectively.

**Table 2:** Performance of the three prediction protocols

Subfamily	HMMER (Sn/Sp)	PSSM-15 (Sn/Sp)	PSSM-44 (Sn/Sp)
AcCS	0.96/1	0.95/1	0.96/1
4CL	0.98/1	0.92/1	0.92/1
LCS	0.98/0.96	0.93/0.96	0.98/0.95
Luciferase	1/1	1/0.99	1/1
MCS	0.91/0.99	0.83/0.97	0.91/0.99
NRPS	0.95/1	0.95/0.98	0.94/0.99
			

**Table 3:** Prediction of substrate preference by whole sequence HMMs and PSSM-15 method for enzymes having anomalous substrate specificity.

Protein	HMMER	PSSM-15
CG6178	Luciferase	Long chain:CoA ligase
At4 g05160 & At5 g63380	Coumarate:CoA Ligase	Long chain:CoA ligase
At1 g62940	Coumarate:CoA ligase	Long chain:CoA ligase

### Prediction of substrate specificity based on SDRs

**Figure 3:**
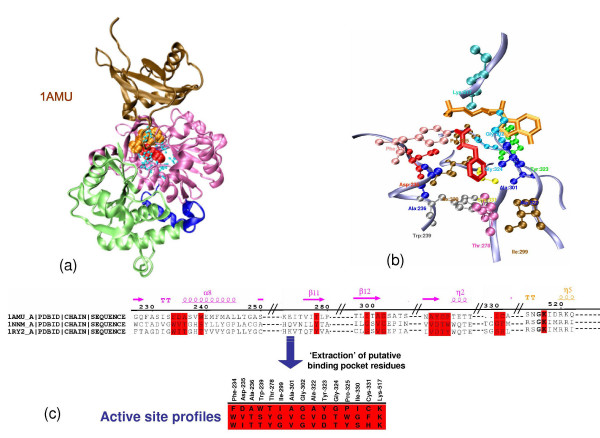
**Identification of Specificity Determining Residues (SDRs)**. (a)The crystal structure of adenylation domain of gramicidin synthetase (PDB code: 1AMU). The C terminal domain is colored in ochre. The A and B subdomains of the N-terminal domain are shown in green and pink respectively. The cofactor AMP (orange) and substrate phenylalanine (red) are depicted as CPK models. The 15 SDRs lining the substrate binding pocket are shown in cyan. (b) Zoomed in version of the active site. The substrate phenylalanine and AMP are shown as stick in red and orange respectively and the 15 SDRs as ball and stick. (c) The extraction of SDRs from a protein sequence. The query sequence is aligned with the structural template and the amino acids of the query corresponding to the SDRs of the template are extracted. The residues highlighted in red represent the SDRs.

It is possible that, the enzymes CG6178, At4 g05160, At5 g63380 and At1 g62940 which show anomalous substrate preference have acquired specificity for long chain substrates as a result of specific mutations in the active site pocket residues, while rest of the protein shows similarity to luciferase or 4CLs. To test the hypothesis, we attempted to develop a prediction protocol based on a limited set of binding pocket residues responsible for substrate selection. In an earlier study on adenylation domains of NRPS, Stachelhaus *et al *have demonstrated that predictions based on binding-pocket residues were significantly better than whole sequence based approaches [32]. Based on the crystal structure of adenylation domain of gramicidin synthetase (PDB code:1AMU) in complex with the substrate phenylalanine, it had been proposed that the substrate selectivity of NRPS adenylation domain is governed by the residues forming the substrate binding pocket [32]. Since the phenylalanine binding site on 1AMU is in close proximity of the conserved AMP binding site and the catalysis by the superfamily of enzymes involve formation of covalent bond between AMP and acyl group, it was reasonable to assume that other subfamilies will have the acyl group binding site at a position structurally analogous to the Phe binding pocket of 1AMU. Therefore in our work, all amino acids which fall within 6 Å distance from the C_β _of the substrate phenylalanine were identified as putative substrate specificity determining residues (SDRs) (Figure 3). By this method, 12 amino acids positions were identified. These include 7 out of the 10 residues which are identified in 1AMU as the substrate binding pocket residues [18]. The remaining three, (position number 239, 278 and 299) were also included in our analysis, even if they were at a distance slightly larger than 6 Å. Hence, a total of 15 amino acid positions were defined as the SDRs. The sequential order of these 15 SDRs was defined as the active site profile (ASP) for each enzyme in the training set (Figure 3). Multiple sequence alignments were obtained for each subfamily by CLUSTALW and separate position specific scoring matrices PSSMs were built for each subfamily as described in methods. The PSSM for the ASPs for each subfamily were used to score the ASP of a query sequence and the substrate preference was assigned based on the highest scoring PSSM. The prediction accuracy of this protocol was also evaluated using the above mentioned test and training set approach. Table 2 also shows the results for prediction of substrate specificity based on 15 active site pocket residues. It was encouraging to note that for the acyl adenylate superfamily, the substrate specificity can also be predicted with high accuracy using a limited number of residues which define the putative substrate binding pocket. The 15 SDRs were able to correctly predict the substrate preference for CG6178 to be long chain:CoA ligase, rather than a luciferase. Similarly for At4 g05160 and At5 g63380 the PSSM-15 based method correctly identified the substrate preference as long chain fatty acid (Table 3). Our SDR profiles indicates that, At1 g62940 is not a 4CL in agreement with experiments. However, by our PSSM-15 based method this sequence was predicted as long chain CoA ligase, rather than medium chain CoA ligase (Table 3). The origin of this discrepancy might be due to the ambiguity in literature regarding definition of acyl chain length which distinguishes medium chain CoA ligase from long chain CoA ligases. Thus, our analysis suggests that, for this superfamily of enzymes a limited number of residues lining the substrate binding pocket are involved in substrate selection. In some cases, the enzymes having whole sequence homology to one subfamily, have evolved to acquire specificity for completely different substrates by mutating a limited number of active site residues. Prediction of substrate specificity based on limited number of specificity determining residues (SDR) has the additional advantage that, based on the identified residues site directed mutagenesis experiments can be designed to alter substrate specificity by protein engineering.

Even though PSSM-15 method could successfully predict for cases in which enzymes showed so called anomalous specificity, the overall prediction accuracy for the PSSM-15 method was slightly lower compared to the prediction by whole sequence HMMs. The prediction accuracy fell from 0.98 to 0.93 for LCS, from 0.91 to 0.83 for MCS and from 0.98 to 0.92 for 4CLs. This could be due to the fact that these 15 SDRs were identified based on a smaller substrate phenylalanine. For larger substrates like luciferases, long chain and medium chain fatty acids the binding pocket could be larger and they can comprise of more number of SDRs. To confirm this hypothesis, SDRs were increased from 15 to 44 by increasing the distance cut-off for defining putative SDRs from 6 Å to 10 Å. The PSSM for each subfamily were rebuilt for 44 positions and the prediction accuracy recalculated. As can be seen, from Table 2 the prediction accuracy with larger binding site was comparable to that obtained by whole sequence based method (HMMER).

### Analysis of conservation pattern of SDRs

**Figure 4:**
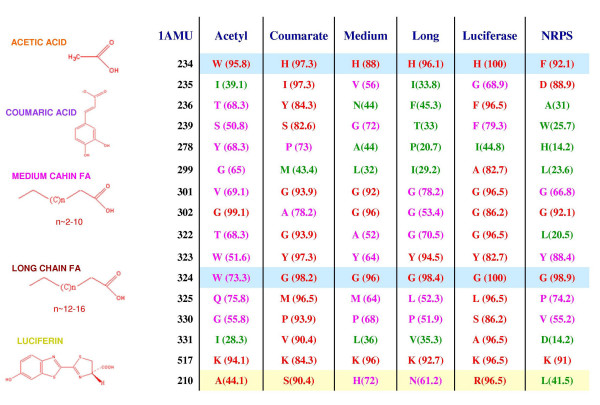
**Consensus Active Site Profile for six subfamilies**. The table lists the conservation pattern of 15 positions that constitute the SDRs for various subfamilies. Number in the bracket refers to the percentage conservation of the amino acid in the alignment. The positions which have conservation >80% or <50% are shown in red and green respectively. Those positions with conservation between 50% and 80% are colored in pink. The positions which are highlighted have a subfamily specific conservation pattern and play a crucial role in controlling substrate specificity. Position number 210 (highlighted in yellow) was identified by docking studies.

Since these 15 residues could successfully predict the substrate preference, it was intriguing to see the amino acid conservation pattern at these positions across the different subfamilies. Figure 4 shows the conservation pattern of the 15 SDR positions (consensus active site profile). The conservation pattern was identified using an approach is similar to that used in the evolutionary trace method, which has been used in earlier studies to identify functional sites on proteins [50]. As can be seen in Figure 4, eight out of these 15 positions clearly change in a subfamily specific manner. The binding pocket of all subfamilies is majorly composed of hydrophobic amino acids. The consensus active site profile of LCS, MCS and luciferases are very similar. This is also reflected in the substrates of the enzymes. LCS and MCS only differ in the chain length of the fatty acid that they use as substrate. There are reports where luciferases have been known to catalyze formation of long chain fatty acyl:CoA [42,51]. Many biochemical and mutagenesis experiments have been carried out to determine the residues governing substrate specificity in this superfamily. These experiments also independently show that, many of these 15 SDRs are indeed important for substrate selection. The position 324 (numbering according to 1AMU) has a conserved tryptophan in AcCS (substrate C_2_), whereas in all other subfamilies which utilize larger substrate (>C_2_), this position has a conserved glycine. Analysis of the substrate binding pocket in the available crystal structures, it clearly shows that bulkier tryptophan makes the substrate binding cavity smaller whereas a glycine will allow accommodating larger substrates (Additional file 1, **Figure S2**). Various structural and mutagenic studies have confirmed the role of tryptophan in controlling the substrate preference of the enzyme [25,38,40,52]. Similarly, position 234 has a conserved tryptophan in AcCS, a conserved histidine in 4CL, LCS, MCS and luciferases and a conserved phenylalanine in NRPSs. We hypothesize that this histidine may play a role in attracting the carboxylic acid substrate into the deep seated binding pocket. In NRPSs this role is played by negatively charged aspartic acid (adjacent position i.e. 235) which interacts with the NH^3+ ^group of amino acids [18]. The conserved tryptophan in AcCS might provide a suitable chemical environment for the substrate acetate which allows it to easily move in and out of the binding pocket. Hence it is hypothesized that this histidine may act as an attracting hook which pulls the larger substrates (like coumaric acid, fatty acid chains, luciferin) into the active site; however for small substrates like acetic acid only chance entrapment can catalyze the reaction. Mutagenic experiments in different subfamilies have shown the importance of this histidine [39,53-56].

### Structural similarity between members of ACS superfamily

**Figure 5:**
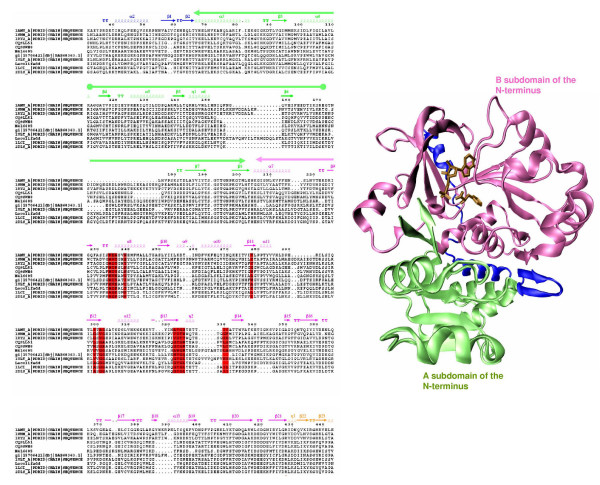
**Structural subdomains of adenylation domain**. The figure depicting the A- (green) and B- (pink) subdomains of the N-terminal domain of 1AMU. The bound AMP and phenylalanine (stick representation) are also shown. The MSA of some of the representative members from each subfamily shows that most of the insertions and deletions are confined to the A-subdomain. The 14 SDRs (highlighted red) belong to the B-subdomain which is relatively conserved.

It is known that, various members of ACS superfamily adopt a highly conserved three dimensional fold despite low sequence similarity. We analyzed the extent of structural similarity between available crystal structures of ACS superfamily of enzymes. Since, the orientation of the C-terminal domain relative to the N-terminal domain is known to change upon ligand binding [26], the structures were aligned only over the N-terminal domain and RMSD was calculated. Simultaneously, the percentage similarity between the sequences is also calculated by Needleman-Wunsch program. The **Table S1 **in Additional file 2summarizes the results. It clearly shows that luciferase (PDB code:1LCI) and acetyl:CoA ligase (PDB code:1PG4) which show only 17% similarity at sequence level, are found to have almost identical folds with RMSD of 1.6 Å. Also luciferases from *Photinus pyralis *(PDB code: 1LCI) and *Luciola cruciata *(PDB code: 2D1R), which show higher similarity (64%) show RMSD in the comparable range (1.1 Å). During the protein structure comparison studies it was noticed that, the large N-terminal domain of the ACS fold, can be further subdivided into two subdomains, namely subdomain A and B. The subdomain A stretches from amino acid 67 to 203 and B subdomain from amino acid 204 to 428 (numbering according to 1AMU). These two domains are relatively independent and there are very few contacts between the two (Figure 5). The B subdomain contains all the substrate binding residues. As seen from the multiple sequence alignment (Figure 5) most of the insertions and deletions are confined to the subdomain A and the subdomain B is relatively more conserved. This may be due to the functional constraint of the superfamily of catalyzing the same reaction but with different substrates. During superimposition the N-terminal domains of various structures of the superfamily, it was found that there are relative movements between the subdomain A and B in different structures. Thus our structural analysis helped in explaining how enzymes of this superfamily can adopt a highly conserved tertiary fold despite large divergence in primary sequence. It is also interesting to note that, the core of the conserved structural fold consists of the subdomain B of the N-terminus domain. Since substrate binding pocket is primarily confined to subdomain B, binding pockets of the various members of this superfamily can be modelled using the limited number of available crystal structures as templates.

### Effect of the choice of structural templates on SDRs

At the time when the work was carried out, crystal structures were available for adenylation domain of NRPS (PDB code:1AMU), luciferases (PDB code:1LCI) and acetyl:CoA ligases (PDB code:1PG4). For long chain:CoA ligases, medium chain CoA ligases and luciferases, ligand free luciferase structure 1LCI was used as the template. However, the SDRs for all subfamilies were identified based on superposition of the modeled structure on 1AMU. With the availability of substrate bound crystal structures for all the subfamilies, it is important to understand whether SDR profiles for various subfamilies would change with the choice of structural template. Currently substrate bound structures are available for five out of the six sub families of acyl adenylate superfamily discussed in this work. They are 1PG3 (acetyl CoA ligase), 3EQ6 (medium chain CoA ligase), 1V26 (long chain CoA ligase), 2D1R (luciferase) and 1AMU (adenylation domain of NRPS). For the enzymes 1PG3, 3EQ6, 1V26 and 2D1R, active site residues were identified by three different methods and compared.

In method 1, the sequences were submitted as query to our web server http://www.nii.res.in/pred_acs_substr.html which models long chain:CoA ligases, medium chain CoA ligases and luciferases using the ligand free luciferase structure 1LCI as the template, but identifies binding pocket residues based on superposition of the modeled structure on 1AMU. In method 2, no structural modelling is carried out, instead the crystal structures 1PG3, 3EQ6 and 1V26 are used, but binding pocket residues are identified based on superposition of the crystal structures on 1AMU. In method 3, binding pocket residues are identified from the ligand bound crystal structures based on amino acids which are within a distance of 6 Å from the ligand. This will correspond to the true binding pocket as seen in experimentally determined structure. The SDRs identified by these three different methods for the four enzymes 3EQ6, 1PG3, 2D1R and 1V26 are listed in Additional file 2, Tables S2-S5.

It is interesting to note that, for 3EQ6, the medium chain CoA ligase 12 out of the 15 binding pocket residues were common in the three different methods for binding pocket identification (Additional file 2, Table S2). The binding pocket in the medium chain fatty acid bound crystal structure had only four additional residues which were not predicted by our web server. Similarly, in case of 1PG3, the acetyl CoA ligase, which has a smaller ligand compared to our reference structure phenylalanine bound 1AMU, 8 out of the 15 binding pocket residues were found to be common in the three different methods and the true binding pocket in the acetyl bound structure had only 2 extra residues (Additional file 2, Table S3). In case of 2D1R, the luciferase, all the 15 amino acids identified by the web server were common to the three different methods, but because of the larger size of luciferin 10 additional residues were identified in the actual luciferin bound crystal structure (Additional file 2, Table S4). Similarly, in case of 1V26, the long chain CoA ligase, 13 out of the 15 binding pocket residues identified by our web server were present in the true binding pocket, but additional residues lined the actual binding pocket in view of the larger chain length of the bound ligand (Additional file 2, Table S5). Thus the binding pocket residues identified based on 1AMU agree very well with the true binding pocket as seen in crystal structure and only in case of enzymes having larger substrates additional residues constitute the binding pocket. In view of the high degree of structural conservation in the superfamily, modeled structures (method 1) and actual crystal structures (method 2) give almost identical binding pocket residues.

Our analysis demonstrates that, in view of the high degree of structural conservation in the acyl adenylate superfamily, active site based substrate specificity can be done using information from single structural template. The identification of SDRs based on single structural template has certain advantages for prediction of substrates for acyl adenylate superfamily without knowing *a priori *the subfamily information. In absence of prior information about subfamily, it will be difficult to choose subfamily specific structural templates. This becomes a major problem, specially in those cases which show anomalous substrate specificities i.e. sequences showing high homology to 4CL but utilizing long chain acyl substrates. Secondly, use of different structural templates from different subfamilies would lead to different number of binding pocket residues and this will make comparison of scores across subfamilies for matches to active site profiles a difficult task. Therefore, in cases where subfamily information is not known, a better approach for prediction of substrate specificity would be would be to first identify the subfamily using single structural template and then align the sequences with the respective templates for more accurate identification of binding pocket residues.

### Ligand docking

**Figure 6:**
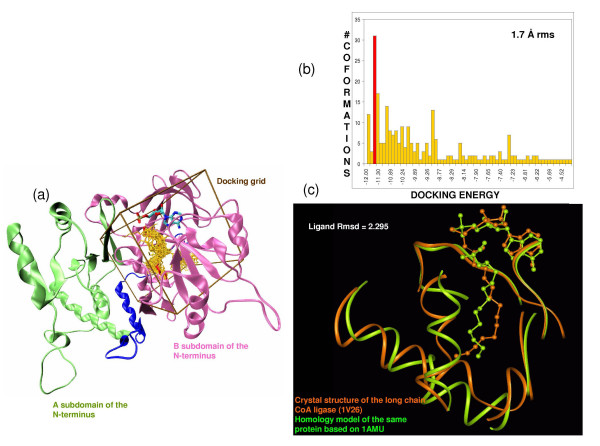
**Docking of myristic acid onto model of long chain:CoA ligase**. (a) The N-terminal domain of the homology model of long chain:CoA liagse. The figure shows that AUTODOCK sampled many conformations (shown in yellow) within the docking grid. (b) Histogram showing the various clusters obtained after docking. The cluster highlighted in red was chosen and the docked conformation was selected. (c) Comparison of the conformation of the docked ligand (green) with the conformation of the same ligand as obtained from X-ray crystallography (PDB code: 1V26) (orange).

We next proceeded to carry out docking of substrates to their respective proteins and understand how exactly the identified SDRs stabilize the cognate substrates in their respective binding sites. In view of the large sequence divergence within each subfamily and availability of only a limited number of crystal structures, structural features of various members of this superfamily can only be obtained by homology modeling approach. We decided to carry out all the docking studies using homology models based on PheA (PDB code:1AMU) and compare the results to actual crystal structures wherever available (Additional file 1, Figure S3). A representative sequence from each subfamily was chosen. Acetyl:CoA ligase from *Salmonella enterica *(GenBank:31616027), long chain:CoA ligase from *Thermus thermophilus *(GenBank:55980573), luciferase from *Photinus pyralis *(GenBank:126501), Coumarate:CoA ligase from *Arabidopsis thaliana *(At4CL2) (GenBank:5702188) and medium chain:CoA ligase from *Pseudomonas oleovorans *(GenBank:416605) were chosen for model building. The structure of adenylation domain of gramicidin synthetase (PDB code:1AMU) was used as a template and its alignments with the query sequence were obtained from GenTHREADER [57]. Based on these alignments MODELLER[58] package was used to build the homology models. During docking simulations the docking grid was defined based on cavities found adjacent to the AMP binding pockets. The cavities were identified in the models, using the program ACSITE [59]. For each model, a docking grid was built to encompass the whole cavity (Figure 6). During docking AMP was kept in the AMP binding site as a fixed ligand.

For AcCS, MCS and LCS, C_2 _(acetic acid), C_10 _(decanoic acid) and C_14 _(myristic acid) fatty acids were used as substrates respectively. At4CL2 has been shown to utilize coumarate and caffeate efficiently [60] and hence caffeate was chosen for docking on the model of At4CL2 (Additional file 1, Figure S3). The conformations of the ligand generated after docking runs were clustered at different RMSD cut off value. For AcCS, this was set at 1 Å and for larger substrates; it was set at 1.7 Å. The final orientation of the ligand which was selected as the docked conformation was based on three criteria; the energy rank, cluster rank and the distance of the carbonyl carbon and the donor phosphate of AMP. The energy rank is based on the docking energy and the cluster rank is based on the number of conformations in the cluster. For the catalytic activity, the carbonyl group of the substrate should be close to the donor oxygen of AMP. Hence, the conformation having lower energy rank and cluster rank and minimum distance between the carbonyl carbon and phosphate of AMP is selected as the docked complex.

**Figure 7:**
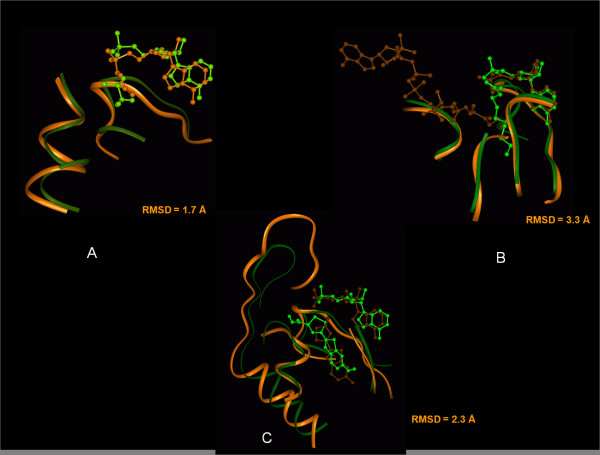
**Comparison of the docking based substrate bound conformations for acetyl CoA synthetase, medium chain CoA ligase and luciferase with the substrate bound structures obtained from X-ray crystallography**. The structures obtained from docking are shown in green, while the corresponding crystal structures are depicted in orange. (A) acetyl CoA ligase (B) medium chain CoA ligase (C) luciferase. The RMSD values shown in the figures correspond to root mean squared deviations in ligand coordinates when the enzyme structures were optimally superposed.

Table 4summarizes the results of docking for each subfamily, while **Figure S4 (A-E) **in Additional file 1shows the final docked orientations for the cognate substrates for each of the different subfamilies. As can be seen from Table 4, except for 4CL, in all other cases the final docked conformation has lower energy as well as cluster rank. Since crystal structure [24] of myristic acid in complex with long chain CoA synthetase was available, we compared it with the docked conformation for long chain fatty acid (C_14_) obtained by our docking simulation. Interestingly, the ligands had a RMSD of 2.2 Å (Figure 6) and 80% of the binding pocket residues identified by docking studies matched with those identified from actual crystal structure. Apart from long chain CoA synthetase, similar comparisons of docked complexes with the available crystal structures of enzyme-substrate complexes were also carried out for acetyl CoA synthetases, luciferase and medium chain CoA ligase. Figure 7 shows the superposition of the docked complexes on to the crystal structures of the corresponding protein-ligand complexes. For acetyl CoA ligase the comparison of the docked complex with the acetyl group bound crystal structure 1PG3 showed that, the ligand had an RMSD of 1.7 Å and 85% of the binding pocket residues in the crystal structure matched with the docked complex (Figure 7A). Figure 7B shows the superposition of the docked complex for medium chain CoA ligase on the medium chain fatty acid bound crystal structure 3EQ6. In our docking studies medium chain CoA ligase from *Pseudomonas oleovorans *(GenBank:416605) was docked with decanoic acid. However, the human medium chain CoA ligase crystal structure (3EQ6) had the substrate butyric acid bound in the active site. The ligands were compared over the first four carbon atoms and the RMSD was 3.3 Å and 78% of the binding pocket residues matched. For luciferase the docked complex was compared with the luciferase from *Luciola cruciata *(PDB code: 2D1R) which had the ligand luciferin bound in the active site. The ligand RMSD was 2.3 Å and 75% of the binding pocket residues matched (Figure 7C).

**Table 4:** Results of docking of various substrates onto their cognate enzymes in each subfamily

Subfamily	**% Iden with **1AMU	Number of docking runs (GALS)	Cluster RMSD (Å)	Docking energy	Energy Rank	Cluster size	Population rank	Distance (Å)
AcCS	18.5	250	1.0	-3.73	4	168	1	3.6
4CL	19.8	250	1.7	-7.74	8	59	2	5.4
MCS	16.3	250	1.7	-8.5	4	6	9	3.1
LCS	18.3	250	1.7	-11.42	3	31	1	4.2
Luciferase	17.7	250	1.7	-11.20	4	61	2	4.3

The table shows the energy and population rank for the cluster having the carboxyl group at interacting distance from the phosphate of AMP. The last column depicts the distance between the carbonyl carbon and oxygen of the donor phosphate.

Hence for this enzyme superfamily, inspite of low sequence similarity, one is able to build approximate homology models, dock the ligand and identify the correct binding pocket. Therefore, the best docking solution from each subfamily was analyzed to identify additional functionally important residues apart from the 15 SDRs. The residue at position number 210 was found to be important for long and medium chain:CoA ligases. The long chain:CoA ligases have a conserved asparagines, whereas MCS have a conserved histidine (Figure 4). This position could potentially be the one controlling substrate chain length. In medium chain:CoA ligases, the presence of a positively charged histidine in the fatty acid binding tunnel will disfavour binding of longer fatty acyl substrates. However, in long chain:CoA ligases, the presence of partially hydrophobic asparagine might favour binding of fatty acids having longer chain length. Interestingly, in luciferases this position has a conserved arginine which has been suggested in earlier studies to form the base of the cavity for luciferin binding [39,40,61].

Thus the results from our docking studies as well as conservation profile analysis of binding pocket residues indicate that, in various subfamilies of acyl adenylate superfamily, the amino acids in the binding pocket have changed collectively to accommodate their respective substrates. The amino acids forming the binding pocket of acetyl:CoA ligases are larger in size than the ones forming the binding pocket of other subfamilies, e.g. position numbers 234, 278, 301, 322. (Figure 4). These observations from our *in silico *analysis are also in agreement with experimental studies. In *Methanothermobacter thermautotrophicus *ACS, the mutation of valine (corresponding to position number 301) to smaller amino acids (alanine) have shown to lengthen the binding pocket to accommodate longer substrates like propionate [52]. The glycine corresponding to position 301 in firefly luciferase (G315) has been found to be very important for proper luciferin binding [40].

### Identifying new members of acyl adenylate superfamily from NR database

**Figure 8:**
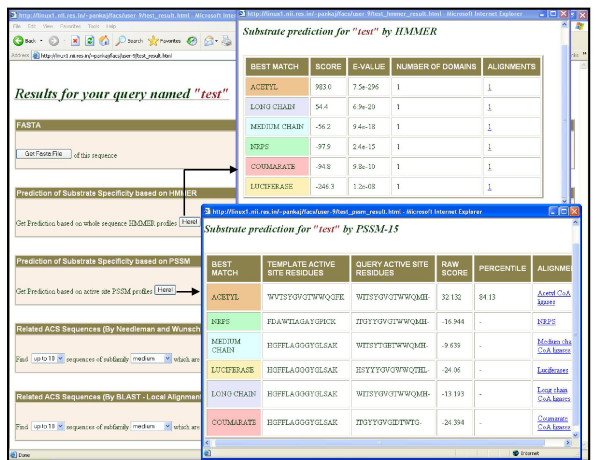
**Typical use of the query interface of pred_acs_substr for analysis of substrate specificity of ACSs**. The prediction of substrate preference of the query sequence is based on two protocols, namely HMMER and PSSM-15. Each method provides the results in a tabular format, which are sorted based on the score of the query sequence against each subfamily. The two results are shown in separate pop-up windows. The last column in each table provides the link to the alignment of the query sequence with the template sequences of each subfamily.

Since the benchmarking studies on the test set of ACS sequences clearly demonstrated the power of SDR based prediction of substrate specificity, we developed a user friendly web based tool for genome scale prediction of substrate preference for acyl CoA synthetase superfamily of enzymes. Figure 8 shows the results of a typical analysis using this web server available at http://www.nii.res.in/pred_acs_substr.html. This server permits classification of acyl AMP superfamily of enzymes into various subfamilies using both whole sequence HMM profile as well as PSSM based on 15 SDRs. We used this novel tool to identify new members of the AMP superfamily from NR database. NR database was downloaded. HMMER profiles built for each subfamily were used to pick up homologous sequences. Duplicated sequences and sequences which were part of the training set were removed. This way a total of 5480 sequences were fetched out. Sequences with length ranging from 300-750 amino acids were considered. Table 5 shows the number of proteins which could be assigned to various substrate specific subfamilies. It is important to note that, using our computational protocol it is possible to assign specificity to a large number of proteins which were originally annotated as just AMP ligases or hypothetical/unknown proteins. Interestingly apart from classifying various AMP ligases as per their chain length specificity, our method has identified several coumarate:CoA ligases and NRPS adenylation domains. These sequences deserve special attention for experimental studies as they do not show obvious sequence homology to coumarate:CoA ligases or NRPS adenylation domains, but might utilize coumaroyl moiety or amino acids as substrates. Thus our analysis demonstrates that, using our computational method it is not only possible to identify ACS superfamily of enzymes in various genomes, but also get valuable clues about the biosynthetic pathways they could possibly be involved based on *in silico *assignment of their substrate specificity.

**Table 5:** Identification and annotation of new members of the acyl-adenylate superfamily.

Subfamily	AMP-ligases (1499)	Hypothetical proteins (235)	Unnamed proteins (64)	Putative proteins (473)	Unknown proteins (5)
AcCS	266	42	21	54	0
4CL	12	7	0	11	1
MCS	151	16	4	23	0
LCS	1065	162	39	341	4
Luciferase	0	0	0	0	0
NRPS	5	8	0	44	0

Total	2276				

The numbers within parenthesis in the top row indicate total number of proteins in NR database which have been originally annotated as acyl-AMP ligases, putative proteins or unknown proteins due to lack of suitable tools for assigning substrate preference. The various rows indicate the number of such proteins which could be grouped into various subfamilies by our substrate specificity prediction methods.

## Conclusions

In this work, we have used an *in silico *approach to investigate the relationship between sequence and substrate specificity for acyl adenylate superfamily of enzymes. We have demonstrated that, using profile HMMs derived from experimentally characterized members of various subfamilies, it is possible to identify cognate substrates for each subfamily with high sensitivity and specificity. However, to alter the substrate utilization profile of an enzyme it is important to identify key residues which might be controlling the substrate preference. Using substrate bound homologous crystal structures, we have identified a limited number of contact residues crucial for substrate recognition i.e. specificity determining residues (SDRs). Benchmarking on a data set of known specificity indicated that, using the SDR patterns the substrate preference can be predicted for different subfamilies with high accuracy. The power of the SDR approach was further demonstrated by correct prediction of substrate in the cases of enzymes which exhibit apparently anomalous substrate preference. Since our substrate specificity prediction method based on SDRs is fast enough for genome scale prediction of substrate preference, we analyzed the nr database of NCBI using this prediction protocol and assigned substrate preference to many uncharacterized sequences.

Molecular modeling of the substrate in the active site of each subfamily was carried out for further understanding the structural basis of substrate preference. Homology models were built for representative member of each family using structural template from a single subfamily and the cognate ligand was docked. The results were compared to actual crystal structures wherever available. It was encouraging to note that despite the low sequence homology, the modeled structures could be superposed on the corresponding crystal structures with a Cα RMSD in the range of 2 to 3 Å. Similarly for the docked LCS-myristic acid complex, the docked ligand could be superposed on the experimentally determined ligand position with an RMSD of 2.2 Å and 80% of the SDRs identified by docking studies matched with those identified from actual crystal structure. Comparison of docked enzyme-substrate complexes involving acetyl CoA liagse, medium chain CoA ligase and luciferase with the corresponding crystal structures showed that, predicted binding sites had reasonably good overlap with the experimentally identified binding sites. In summary, our structural analysis helped in explaining how enzymes of this superfamily can adopt a highly conserved tertiary fold despite large divergence in primary sequence. The core of the conserved structural fold consists of the subdomain B of the N-terminus domain, while most insertions and deletions in primary sequence are confined to the subdomain A. Since substrate binding pocket is primarily confined to subdomain B, binding pockets of the various members of this superfamily can also be modelled using the limited number of available crystal structures as templates, despite high sequence divergence.

## Methods

### Compilation of the sequences

Since, sequences with experimentally confirmed specificities were crucial for our analysis the dataset had to be curated carefully. All the sequences of adenylation domain of NRPSs were extracted from NRPSDB http://www.nii.res.in/nrps-pks.html[34] which contains manually curated substrate specificities for adenylation domains based on experimentally characterized NRPS gene clusters. Since adenylation domains often occur in multiple copies on a single polypeptide chain of a multifunctional enzyme, it was easy to obtain confirmed specificities of a large number of NRPS adenylation domains based on experimentally identified chemical structure of the nonribosomal peptide product. However, for other families like 4CL, luciferase, acetyl CoA ligase, medium chain CoA ligase and long chain CoA ligase such large numbers of experimentally characterized sequences were not available. Based on extensive literature survey we collected at least 20 biochemically characterized sequences for AcCS, LCS, 4CL and at least 10 characterized sequences for MCS and luciferases. Since, such small numbers of sequences are not adequate for HMM and PSSM analysis using a training and test set approach, we had to include additional sequences in each group using the experimentally characterized sequences as seed sequence. The additional members were selected based on the criteria of low e-value, alignment over the whole length, and same annotation as the seed sequence. Finally, a data set comprising of 608 protein sequences with known substrate specificity (120 AcCS, 130 LCS, 119 4CL, 25 luciferases and 29 MCS) were collected by keyword search and repeated BLAST searches[47] against the NR database of NCBI [48].

### Sequence analysis

Pairwise sequence comparisons were carried out by Needleman and Wunsch alignment program of the EMBOSS package [62]. BLAST was used to perform local alignment [63]. BLOSUM62 scoring matrix and default values for gap penalties were used for sequence alignments. Multiple alignments and phylogenetic dendrograms were also constructed for each of the domain types using CLUSTALW program [64].

### Building PSSM

For building the PSSM, for each subfamily columns corresponding to 15 active site positions were extracted from the alignment. The score of amino acid 'a' at column/position 'p' is defined as:

Freq(a, p) refers to the frequency of residue 'a' at position 'p' in the alignment. 0.05 represents the probability of random occurrence of any amino acid.

If any amino acid occurs with frequency of '0', then a large fixed negative score of -3.00 is assigned to the amino acid at that position. Hence for each subfamily a 15 × 21 position specific scoring matrix (PSSM) (corresponding to 15 positions and 20 amino acids + gap) was calculated, which quantifies the occurrence of amino acids at each position.

### Calculation of prediction accuracy

For the data which are predicted as positive, the actual positive ones are called true positives (TP), while the others are called false positives (FP). For the data which are predicted as negative, the actual positive ones are called false negatives (FN), while the others are called true negatives (TN). The sensitivity and specificity are defined as:-

### Structure analysis

For model building, the alignments with the template structure 1AMU were generated by program GenTHREADER [57]. The models of proteins were built by MODELLER 6V2[58]. The model structures were optimized by conjugate gradient energy minimization and molecular dynamics with simulated annealing provided in the MODELLER program. The ligands for docking were built using the builder module of InsightII. The ligands were minimized before docking using the discover module of InsightII.

### Protein ligand docking

Dockings were carried out by molecule docking evaluation program, AUTODOCK 3.0 [65]. Dockings were done by Lamarckian genetic algorithm. Each docking experiment consisted of a series of 100 simulations, each producing a docking solution. The docking experiment was performed 250 times to give 250 possible solutions. The solutions having an RMSD over all atoms of < 1 Å (for small substrate) or <1.7 Å (for large substrate) were grouped together in a single cluster. Parameters for the docking used were: a population size of 50, a random starting position and conformation, a maximal mutation of 2 Å in translation and 50° in rotations, an elitism of 1, a mutation rate of 0.02, a crossover rate of 0.8 and a local search rate of 0.06. Simulations were performed with a maximum of 1.5 million energy evaluations and 27000 generations. The center of the ligand was chosen as the root. The 60 × 60 × 60 grid with grid points separated by 0.375 Å was build centered on the ligand.

## Authors' contributions

PK performed the computations, analyzed data and wrote the manuscript; RSG and DM designed research, analyzed data and wrote the manuscript. All the authors read and approved the final manuscript.

## Supplementary Material

Additional file 1 **Supplementary figures**. Supplementary figures S1, S2, S3, S4A-S4E. Click here for file

Additional file 2 **Supplementary tables**. Supplementary tables S1, S2, S3, S4, S5. Click here for file
