# Supplementary material for: Genome scale prediction of substrate specificity for acyl adenylate superfamily of enzymes based on active site residue profiles
Source: BMC Bioinformatics. 2010 Jan 27;11:57. doi: 10.1186/1471-2105-11-57 (PMC3098103; doi:10.1186/1471-2105-11-57)
Supplement: Additional file 1 — Supplementary figures. Supplementary figures S1, S2, S3, S4A-S4E. [file 1471-2105-11-57-S1.DOC]

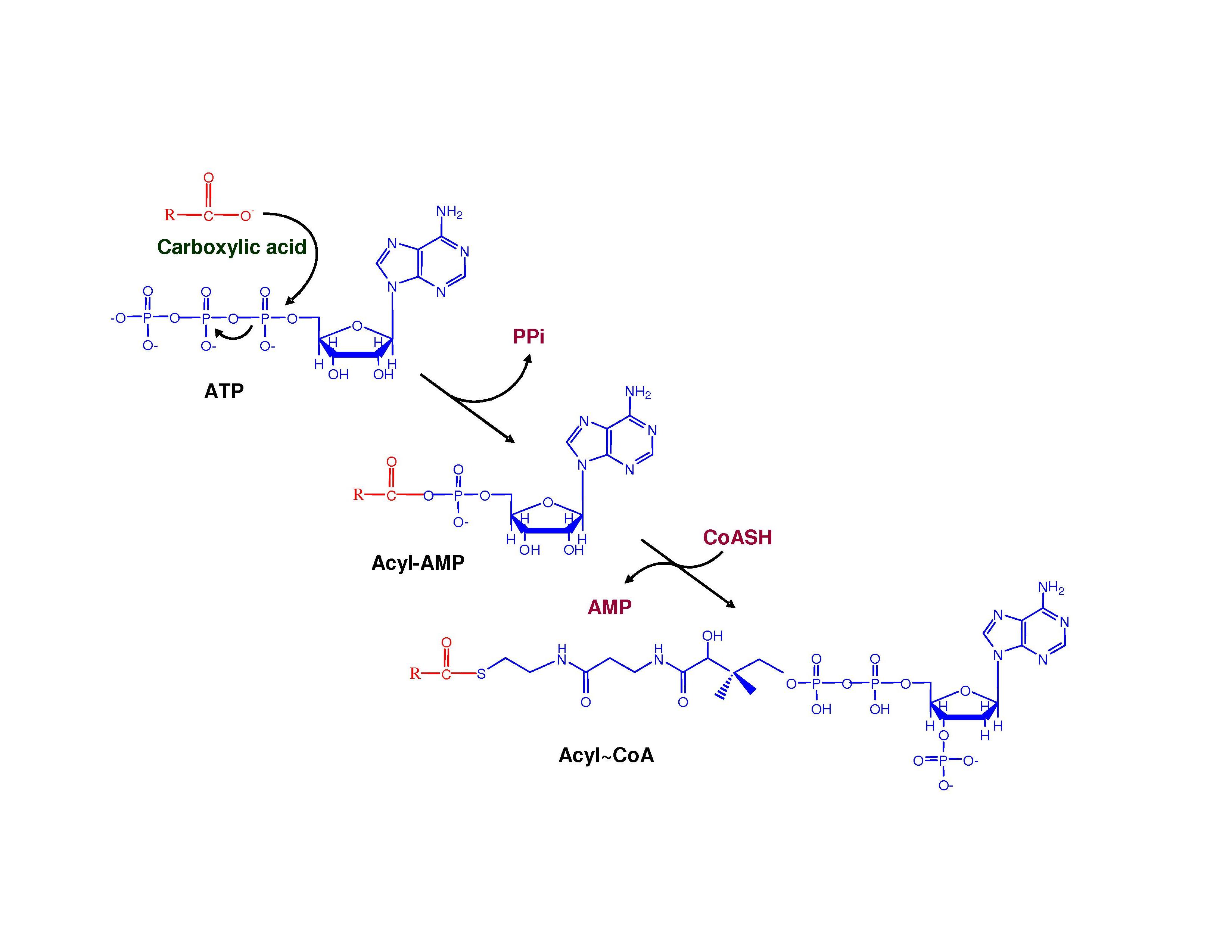


## Figure S1

Schematic representation depicting various steps of the reaction catalyzed by Acyl-CoA Synthetases.


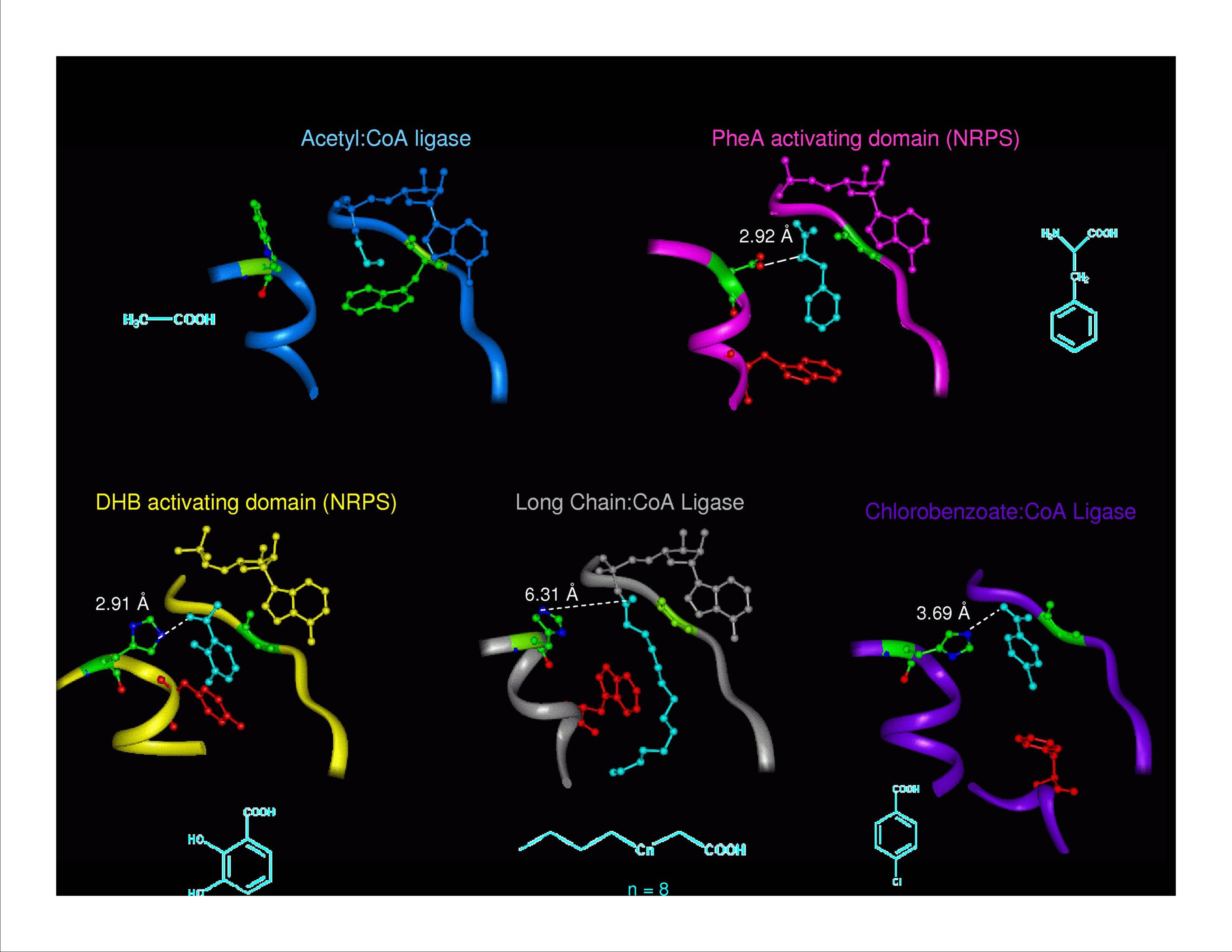


## Figure S2:

The figure showing crucial specificity determining residues in the active site pockets of acetyl:CoA ligase (PDB code1PG4), phenylalanine activating domain of gramicidin synthetase (PDB code:1AMU), DHB activating domain (PDB code:1MD9), long chain:CoA ligase (PDB code:1V26), chlorobenzoate:CoA ligase (PDB code:1T5D).


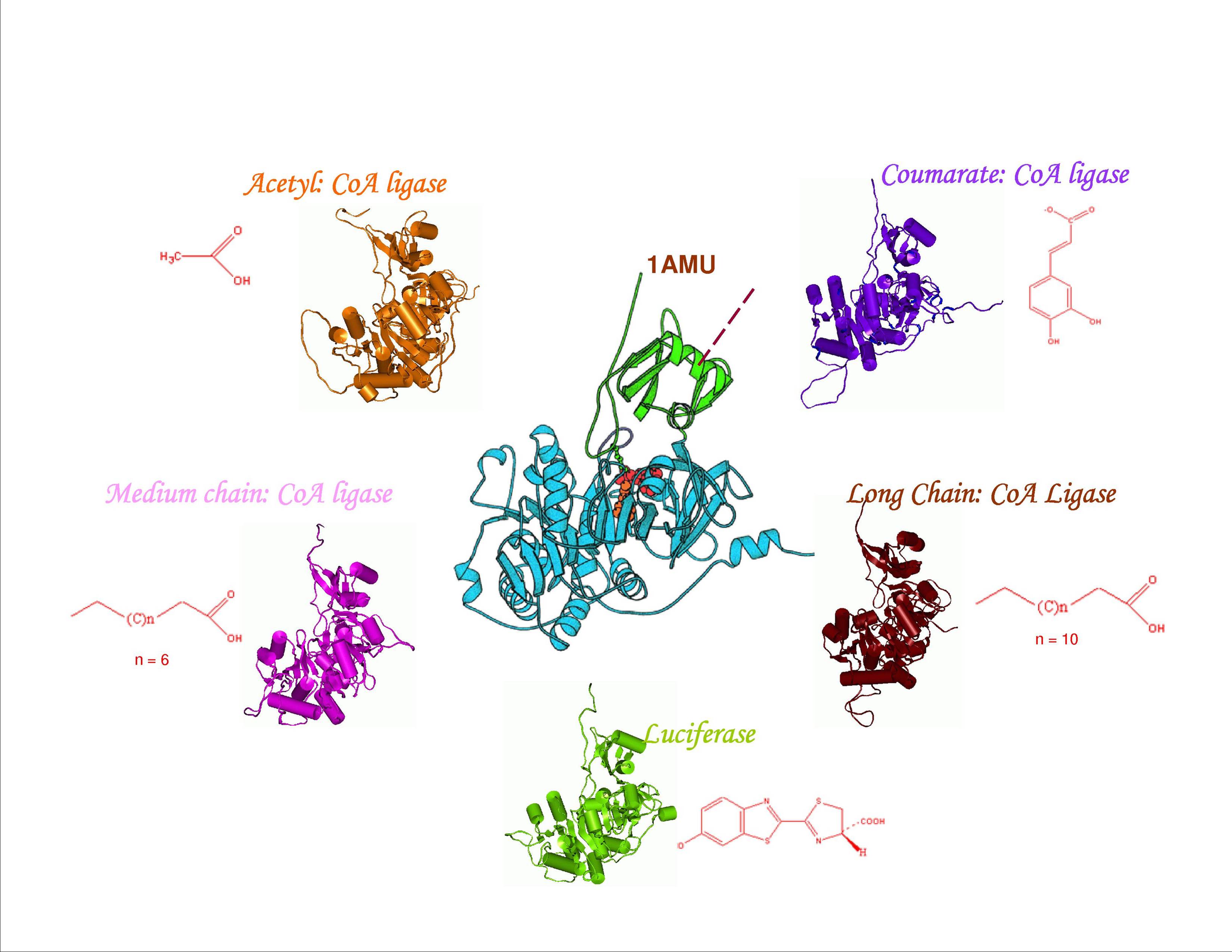


## Figure S3:

Homology models used in the docking simulations for representative member of each subfamily and their cognate substrates.


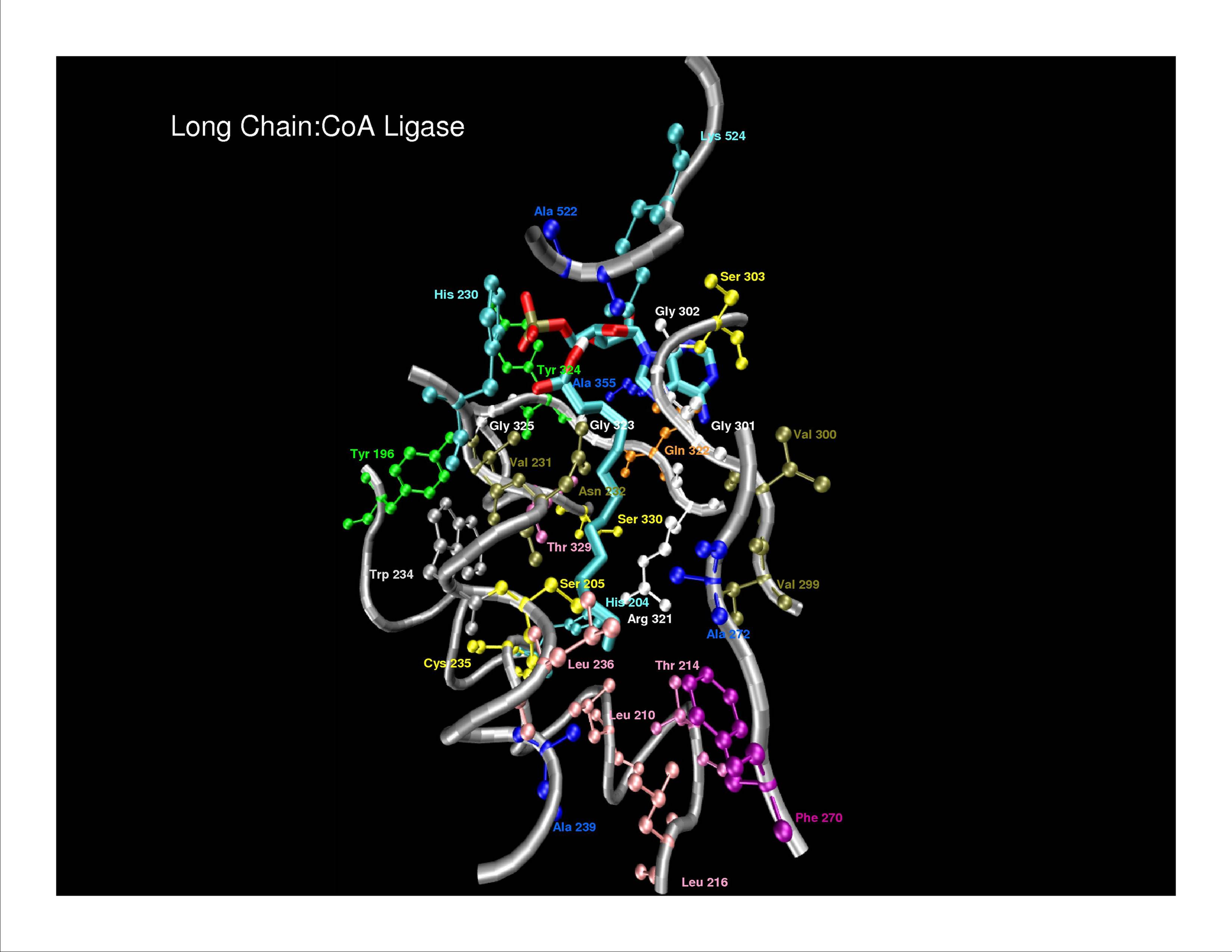


## Figure S4A:

The docked conformations of ligands in the active site pocket of Long chain:CoA ligase. The cofactor AMP and the substrate carboxylic acid is drawn as sticks and colored according to atom type. The residues forming the putative substrate binding pocket are drawn as CPK and colored according to residue type.


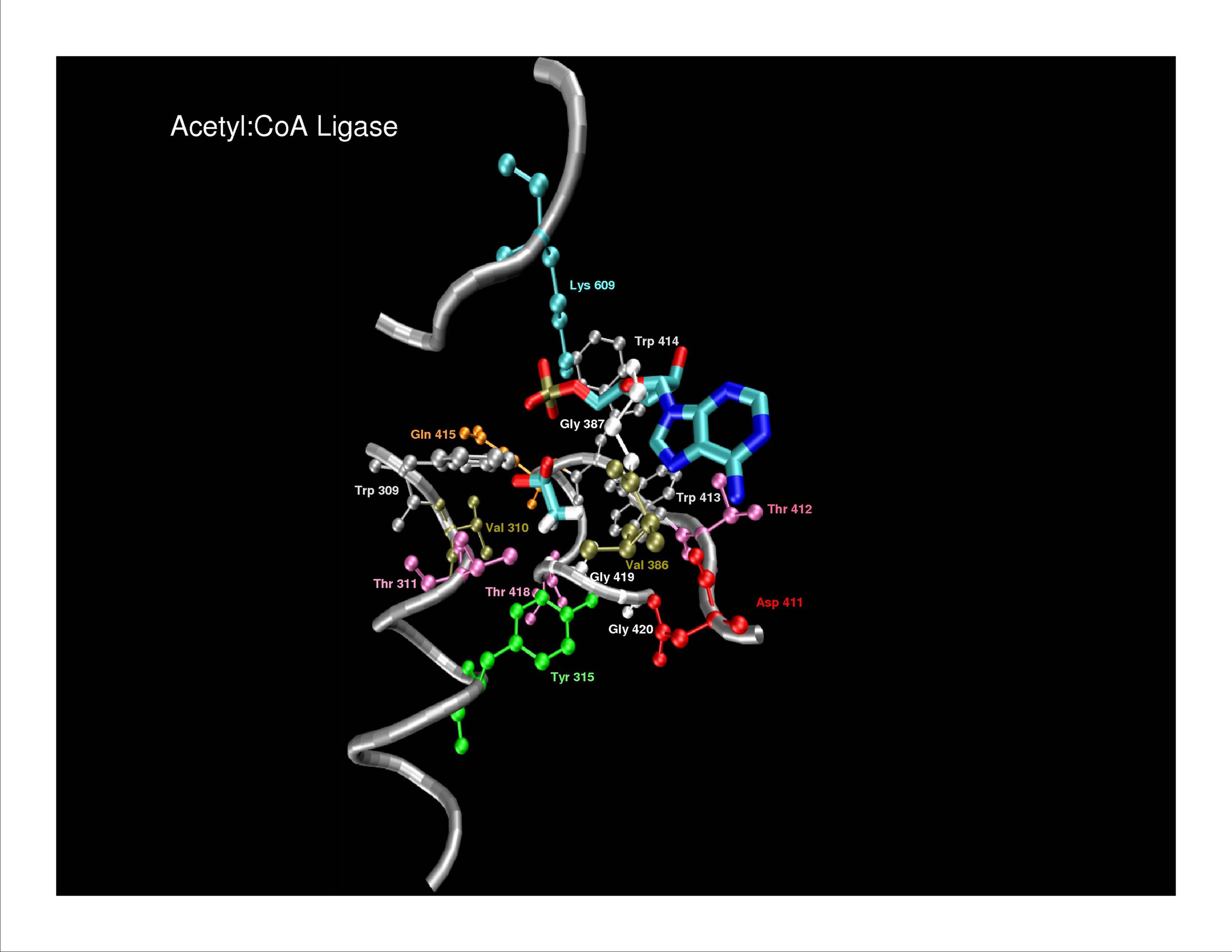


## Figure S4B:

The docked conformations of ligands in the active site pocket of Acetyl:CoA ligase. The cofactor AMP and the substrate carboxylic acid is drawn as sticks and colored according to atom type. The residues forming the putative substrate binding pocket are drawn as CPK and colored according to residue type.


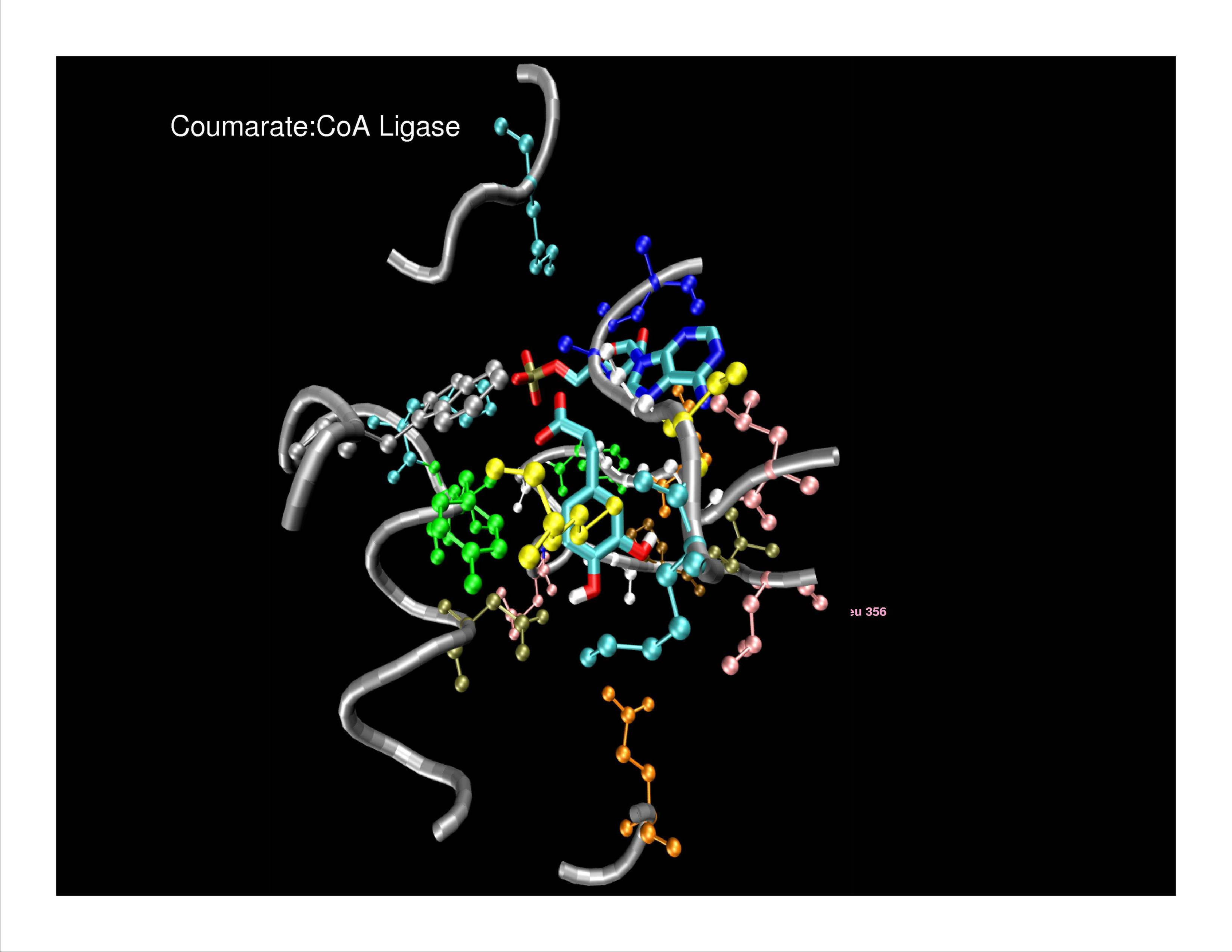


## Figure S4C:

The docked conformations of ligands in the active site pocket of Coumarate:CoA ligase. The cofactor AMP and the substrate carboxylic acid is drawn as sticks and colored according to atom type. The residues forming the putative substrate binding pocket are drawn as CPK and colored according to residue type.


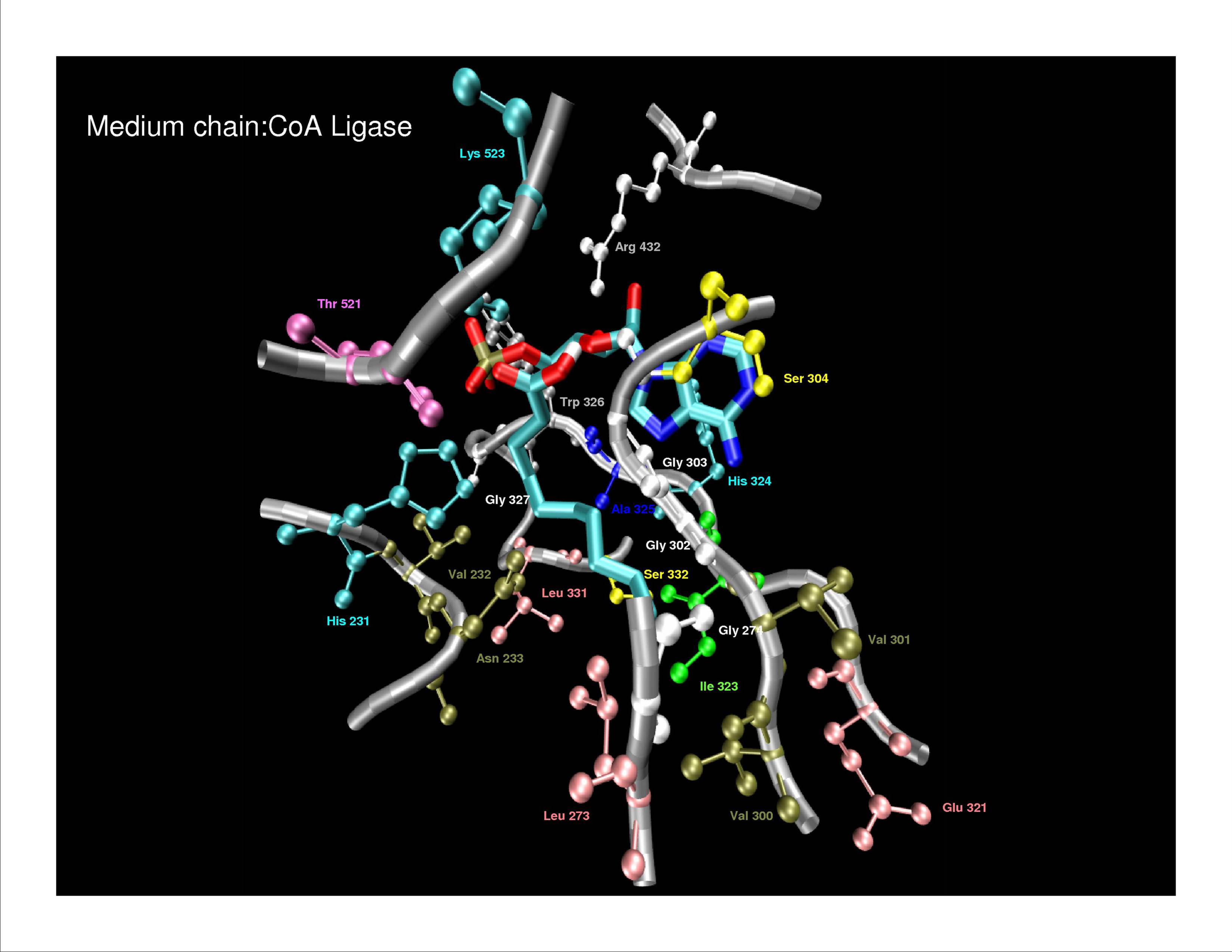


## Figure S4D:

The docked conformations of ligands in the active site pocket of Medium chain: CoA ligase. The cofactor AMP and the substrate carboxylic acid is drawn as sticks and colored according to atom type. The residues forming the putative substrate binding pocket are drawn as CPK and colored according to residue type.


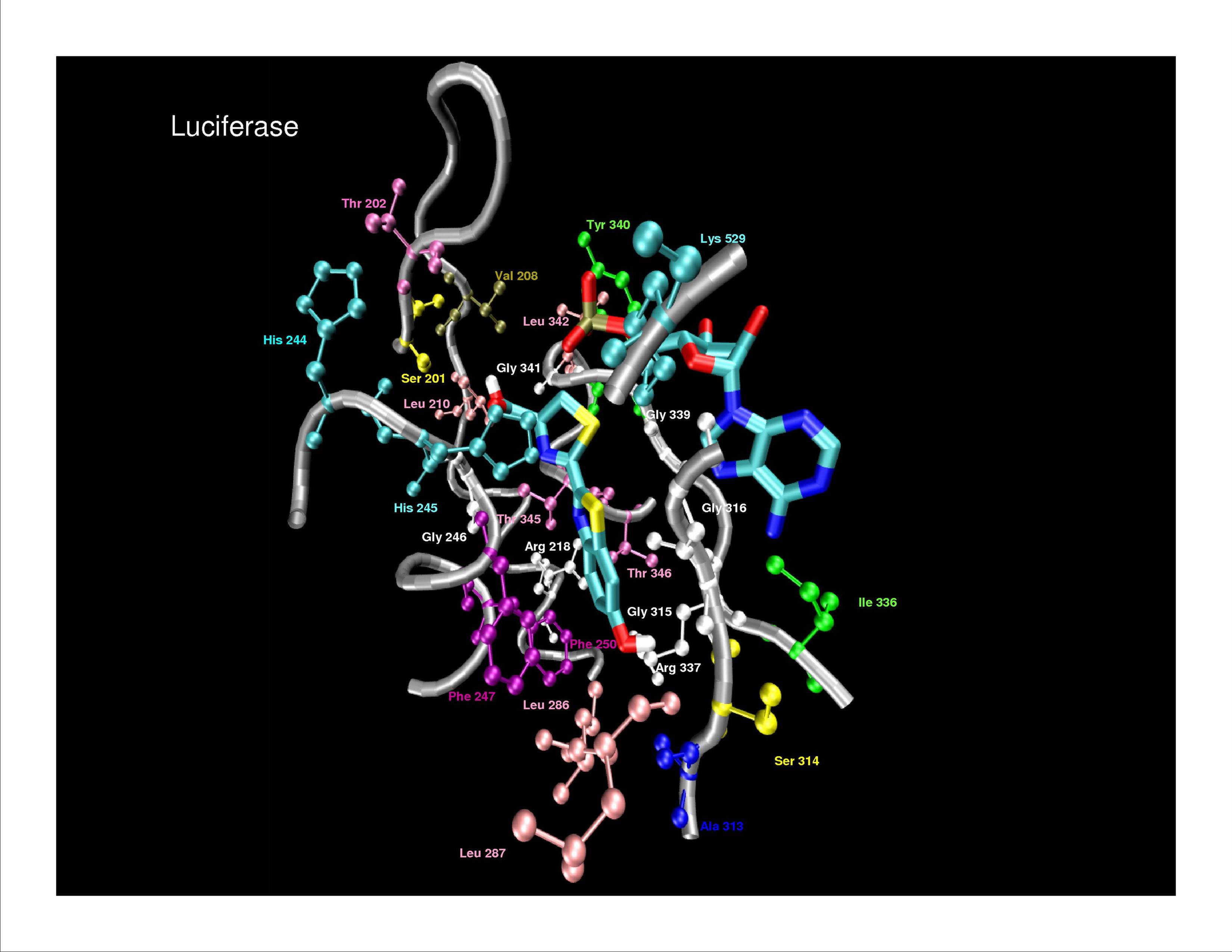


## Figure S4E:

The docked conformations of ligands in the active site pocket of Luciferase. The cofactor AMP and the substrate carboxylic acid is drawn as sticks and colored according to atom type. The residues forming the putative substrate binding pocket are drawn as CPK and colored according to residue type.
