# Supplementary material for: Genome scale prediction of substrate specificity for acyl adenylate superfamily of enzymes based on active site residue profiles
Source: BMC Bioinformatics. 2010 Jan 27;11:57. doi: 10.1186/1471-2105-11-57 (PMC3098103; doi:10.1186/1471-2105-11-57)
Supplement: Additional file 2 — Supplementary tables. Supplementary tables S1, S2, S3, S4, S5. [file 1471-2105-11-57-S2.DOC]

**Table S1: High structural conservation in spite of low sequence similarity**

|  | **1AMU** | **1PG3** | **1RY2** | **1MD9** | **1LCI** | ***1V26*** | **1T5D** | **2D1R** | **2VSQ** | **3DHV** | **3G7S** | **3CW8** | **3EQ6** | **2V7B** | **3IVR** | **3E53** |
| --- | --- | --- | --- | --- | --- | --- | --- | --- | --- | --- | --- | --- | --- | --- | --- | --- |
| **1AMU** |  | 19 | 18 | 22 | 20 | 21 | 20 | 20 | 34 | 26 | 20 | 21 | 19 | 22 | 17 | 23 |
| **1PG3** | 1.7 |  | 43 | 20 | **17** | 20 | 18 | 19 | 21 | 16 | 20 | 19 | 23 | 23 | 21 | 17 |
| **1RY2** | 1.7 | 0.9 |  | 19 | 20 | 19 | 21 | 20 | 19 | 17 | 18 | 22 | 25 | 22 | 19 | 23 |
| **1MD9** | 1.6 | 1.6 | 1.6 |  | 23 | 29 | 23 | 24 | 24 | 22 | 23 | 24 | 21 | 23 | 28 | 20 |
| **1LCI** | 1.6 | **1.6** | 1.6 | 1.7 |  | 23 | 22 | **64** | 21 | 21 | 23 | 22 | 20 | 24 | 23 | 23 |
| **1V26** | 1.6 | 1.7 | 1.7 | 1.5 | 1.7 |  | 24 | 25 | 24 | 18 | 26 | 25 | 20 | 27 | 27 | 22 |
| **1T5D** | 1.6 | 1.6 | 1.6 | 1.7 | 1.6 | 1.6 |  | 21 | 24 | 21 | 23 | 99 | 20 | 23 | 27 | 21 |
| **2D1R** | 1.7 | 1.7 | 1.7 | 1.7 | **1.1** | 1.6 | 1.7 |  | 24 | 22 | 26 | 21 | 20 | 22 | 24 | 20 |
| **2VSQ** | 1.1 | 1.7 | 1.6 | 1.7 | 1.6 | 1.6 | 1.6 | 1.8 |  | 27 | 21 | 24 | 19 | 25 | 24 | 22 |
| **3DHV** | 1.4 | 2.0 | 1.7 | 1.7 | 1.6 | 1.6 | 1.7 | 1.6 | 1.5 |  | 22 | 22 | 21 | 23 | 24 | 23 |
| **3G7S** | 1.5 | 1.6 | 1.6 | 1.5 | 1.5 | 1.6 | 1.6 | 1.5 | 1.6 | 1.6 |  | 23 | 20 | 20 | 25 | 19 |
| **3CW8** | 1.6 | 1.6 | 1.6 | 1.6 | 1.6 | 1.6 | 0.4 | 1.7 | 1.6 | 1.6 | 1.5 |  | 20 | 24 | 27 | 21 |
| **3EQ6** | 1.6 | 1.5 | 1.5 | 1.6 | 1.6 | 1.6 | 1.7 | 1.7 | 1.5 | 1.6 | 1.4 | 1.6 |  | 22 | 23 | 23 |
| **2V7B** | 1.5 | 1.4 | 1.5 | 1.4 | 1.7 | 1.4 | 1.5 | 1.6 | 1.6 | 1.5 | 1.4 | 1.5 | 1.5 |  | 26 | 23 |
| **3IVR** | 1.4 | 1.6 | 1.6 | 1.7 | 1.6 | 1.5 | 1.6 | 1.7 | 1.4 | 1.6 | 1.5 | 1.6 | 1.6 | 1.5 |  | 23 |
| **3E53** | 1.5 | 1.6 | 2.0 | 1.6 | 1.7 | 1.6 | 1.6 | 1.7 | 1.5 | 1.6 | 1.6 | 1.6 | 1.6 | 1.5 | 1.5 |  |

The sequence similarity between various crystal structures of ACS superfamily and their structural similarity (ProFit RMSD) as calculated over the N-terminal domain.

**Table S2: Comparison of SDRs of Medium chain:CoA ligase (PDB id 3EQ6)**

| 1AMU | Method 1  (Web server) | Method 2  (Structural superposition of crystal structure on 1AMU) | Method 3  (Contact residues from substrate bound complex ) |
| --- | --- | --- | --- |
| 234 Phe | 265 Trp | 265 Trp | 265 Trp |
| 235 Asp | 266 Ile | 266 Ile | 266 Ile |
| 236 Ala | 267 Leu | 267 Leu | 267 Leu |
| 239 Trp | 270 Leu | 270 Leu | 270 Leu |
| 278 Thr | 309 Met | 309 Met |  |
| 299 Ile | 335 Val | 335 Val |  |
| 301 Ala | 337 Val | 337 Val | 337 Val |
| 302 Gly | 338 Gly | 338 Gly | 338 Gly |
| 322 Ala | 360 Ser | 360 Ser |  |
| 323 Tyr | 361 Tyr | 361 Tyr | 361 Tyr |
| 324 Gly | 362 Gly | 362 Gly | 362 Gly |
| 325 Pro | 363 Gln | 363 Gln | 363 Gln |
| 330 Ile | 368 Leu | 368 Leu | 368 Leu |
| 331 Cys | 369 Thr |  |  |
| 517 Lys | 557 Lys | 557 Lys |  |
|  |  |  |  |
| Additional Residues |  |  |  |
|  |  | 367 Gly | 367 Gly |
|  |  |  | 339 Glu |
|  |  |  | 364 Thr |
|  |  |  | 366 Thr |
|  |  |  | 470 Gly |

The table shows the position numbers and amino acids for the 15 SDRs in 1AMU and the SDRs for Medium chain CoA ligase extracted by three different methods.

**Table S3:** **Comparison of SDRs of Acetyl chain:CoA ligase (PDB id 1PG3)**

| 1AMU | Method 1  (Web server) | Method 2  (Structural superposition of crystal structure on 1AMU) | Method 3  (Contact residues from substrate bound complex ) |
| --- | --- | --- | --- |
| 234 Phe | 309 Trp | 309 Trp |  |
| 235 Asp | 310 Val | 310 Val | 310 Val |
| 236 Ala | 311 Thr | 311 Thr | 311 Thr |
| 239 Trp | 314 Ser | 314 Ser |  |
| 278 Thr | 355 Tyr | 355 Tyr |  |
| 299 Ile | 384 Gly | 384 Gly |  |
| 301 Ala | 386 Val | 386 Val | 386 Val |
| 302 Gly | 387 Gly | 387 Gly | 387 Gly |
| 322 Ala | 412 Thr | 412 Thr | 412 Thr |
| 323 Tyr | 413 Trp | 413 Trp | 413 Trp |
| 324 Gly | 414 Trp | 414 Trp | 414 Trp |
| 325 Pro | 415 Gln | 415 Gln | 415 Gln |
| 330 Ile | 420 Gly | 420 Gly |  |
| 331 Cys | 421 Phe | 421 Phe |  |
| 517 Lys | 609 Lys | 609 Lys |  |
|  |  |  |  |
| Additional residues |  |  |  |
|  |  |  | 416 Thr |
|  |  |  | 524 Gly |

The table shows the position numbers and amino acids for the 15 SDRs in 1AMU and the SDRs for acetyl CoA ligase (PDB id. 1PG3) extracted by three different methods.

**Table S4:** **Comparison of SDRs of Luciferase (PDB id 2D1R)**

| 1AMU | Method 1  (Web server) | Method 2  (Structural superposition of crystal structure on 1AMU) | Method 3  (Contact residues from substrate bound complex ) |
| --- | --- | --- | --- |
| 234 Phe | 247 His | 247 His | 247 His |
| 235 Asp | 248 Gly | 248 Gly | 248 Gly |
| 236 Ala | 249 Phe | 249 Phe | 249 Phe |
| 239 Trp | 252 Phe | 252 Phe | 252 Phe |
| 278 Thr | 288 Ile | 288 Ile | 288 Ile |
| 299 Ile | 315 Ala | 315 Ala | 315 Ala |
| 301 Ala | 317 Gly | 317 Gly | 317 Gly |
| 302 Gly | 318 Gly | 318 Gly | 318 Gly |
| 322 Ala | 341 Gly | 341 Gly | 341 Gly |
| 323 Tyr | 342 Tyr | 342 Tyr | 342 Tyr |
| 324 Gly | 343 Gly | 343 Gly | 343 Gly |
| 325 Pro | 344 Leu | 344 Leu | 344 Leu |
| 330 Ile | 349 Ser | 349 Ser | 349 Ser |
| 331 Cys | 350 Ala | 350 Ala | 350 Ala |
| 517 Lys | 531 Lys | 531 Lys | 531 Lys |
|  |  |  |  |
| Additional residues |  |  |  |
|  |  |  | 201 Ser |
|  |  |  | 220 Arg |
|  |  |  | 253 Thr |
|  |  |  | 316 Ser |
|  |  |  | 339 Arg |
|  |  |  | 340 Gln |
|  |  |  | 345 Thr |
|  |  |  | 348 Thr |
|  |  |  | 353 Ile |
|  |  |  | 529 Thr |

The table shows the position numbers and amino acids for the 15 SDRs in 1AMU and the SDRs for luciferase (PDB id. 2D1R) extracted by three different methods.

**Table S5:** **Comparison of SDRs of long chain:CoA ligase (PDB id 1V26)**

| 1AMU | Method 1  (Web server) | Method 2  (Structural superposition of crystal structure on 1AMU) | Method 3  (Contact residues from substrate bound complex ) |
| --- | --- | --- | --- |
| 234 Phe | 230 His | 230 His |  |
| 235 Asp | 231 Val | 231 Val | 231 Val |
| 236 Ala | 232 Asn |  |  |
| 239 Trp | 235 Cys | 235 Cys | 235 Cys |
| 278 Thr | 272 Ala | 272 Ala | 272 Ala |
| 299 Ile | 299 Val | 299 Val | 299 Val |
| 301 Ala | 301 Gly | 301 Gly | 301 Gly |
| 302 Gly | 302 Gly | 302 Gly | 302 Gly |
| 322 Ala | 323 Gly | 323 Gly | 323 Gly |
| 323 Tyr | 324 Tyr | 324 Tyr | 324 Tyr |
| 324 Gly | 325 Gly | 325 Gly | 325 Gly |
| 325 Pro | 326 Leu | 326 Leu | 326 Leu |
| 330 Ile | 331 Pro | 331 Pro | 331 Pro |
| 331 Cys | 332 Val | 332 Val | 332 Val |
| 517 Lys | 524 Lys | 524 Lys |  |
|  |  |  |  |
| Additional residues |  |  |  |
|  |  | 233 Ala | 233 Ala |
|  |  |  | 204 His |
|  |  |  | 205 Ser |
|  |  |  | 208 Ala |
|  |  |  | 209 Ser |
|  |  |  | 214 Thr |
|  |  |  | 216 Leu |
|  |  |  | 234 Trp |
|  |  |  | 236 Leu |
|  |  |  | 239 Ala |
|  |  |  | 270 Phe |
|  |  |  | 300 Val |
|  |  |  | 303 Ser |
|  |  |  | 322 Gln |
|  |  |  | 327 Thr |
|  |  |  | 334 Val |
|  |  |  | 335 Gln |
|  |  |  | 439 Lys |
|  |  |  | 444 Trp |

The table shows the position numbers and amino acids for the 15 SDRs in 1AMU and the SDRs for long chain CoA ligase (PDB id. 1V26) extracted by three different methods.
